# Weighing density and kinship: Aggressive behavior and time allocation in fire salamander (*Salamandra infraimmaculata*)

**DOI:** 10.1371/journal.pone.0220499

**Published:** 2019-08-05

**Authors:** Daniel Berkowic, Shai Markman

**Affiliations:** Department of Biology & Environment, Faculty of Natural Sciences, University of Haifa–Oranim, Tivon, Israel; Universidade Federal de Uberlândia, BRAZIL

## Abstract

Kin-biased behavior (that is responding differentially to kin and non-kin) is thought to be adaptive in many social interactions. One example of this kin bias is behaving less aggressively toward a relative than a non-relative, a behavior which yields inclusive fitness benefits. However, data are lacking about the ability of animals to weigh their preference for kinship and the density of conspecifics simultaneously and to respond accordingly. Fire salamanders (*Salamandra infraimmaculata*) larviposit in high densities in ponds. Thus, larvae of different females confront competition and predation by other larvae. We studied whether larvae prefer their kin over particular density or vice versa. We experimentally used a transparent glass aquarium with inner chambers to test the responses of a focal larva toward its siblings and non-siblings. Specifically, we quantified the time a focal larva spent near its siblings or non-siblings, presented in varying densities, and the aggression level it demonstrated. We found that focal larvae spent more time near non-siblings if non-sibling and sibling groups were of equal density. The focal larvae were also more aggressive toward non-siblings. The results may be explained by the cannibalistic nature of these larvae: high density may provide more opportunities for food, especially when non-siblings are present. Further explanations for these findings may include other advantages of staying in a larger group and/or the stronger olfactory and visual stimulation offered by groups compared to a single individual. These findings suggest that larvae make differential responses toward conspecifics depending simultaneously on the level of relatedness and the density of the group. Such responses have important implications for social—aggregation decisions and may especially affect the fitness of cannibalistic species.

## Introduction

Behavioral reactions toward conspecifics based on the ability to recognize kin may be adaptive in some social contexts [1–3]. Advantages associated with such reactions include minimizing inbreeding events, promoting outbreeding [4–7], and decreasing aggression toward relatives, including reducing cannibalism [8, 9].

Kin-biased behavior confers advantages as predicted by kin-selection theory, which suggests that an organism will pursue strategies that favor the reproductive success or survival of its relatives, even at a cost to the organism's own survival and reproduction [10–12]. Therefore, if kin recognition reduces aggression toward relatives, staying near relatives may reduce aggression among unrelated conspecifics, which could potentially increase the fitness of individuals.

However, in general, even when there are no relatives nearby, there might be advantages to joining a larger group of conspecifics, especially when there are no or low costs and the benefits exceed the costs [13]. These advantages may include the effects of dilution, due to the lower likelihood of being preyed upon if an individual joins a larger group, and predator confusion, whereby prey behaviors make it harder for a predator to focus on a particular prey item [14]. Further benefits to joining a larger group include effective foraging (when group foraging is more productive) [14], higher vigilance by all group members, and the creation of information centers that may lead more individuals to better foraging sites [15].

Studies focusing on sibling recognition in amphibians demonstrate that certain salamander and anuran species can distinguish between siblings and non-siblings [2, 3, 16–17]. Such abilities of distinction in cannibalistic salamanders like the marbled salamander *Ambystoma opacum* are important for decreasing aggression and cannibalism among siblings [8, 16].

The fire salamander (*Salamandra infraimmaculata*) inhabits the mountains of northern Israel, such as Mount Carmel and those in the Lower Galilee [18]. Fire salamander larvae feed on insects, crustaceans, and amphibians [19]. Fire salamanders larviposit in high densities in ponds. The period when female fire salamanders larviposit is critical because the first cohort may be in danger of ponds drying out because of unpredictable precipitation, while the last cohort may encounter dry conditions in late spring and early summer [20, 21]. Fire salamanders typically larviposit in ponds that already contain larvae of different females [22]. Thus, young larvae may face other larvae of different genetic relatedness in the pool.

Larvae may experience differential levels of intraspecific aggression that range from non-harmful bites to cannibalistic acts [23]. The fitness of the assaulted larvae may be reduced if the wounds and/or energy spent to escape the aggression affect their foraging abilities, development, or vulnerability to pathogens [23]. Thus, choosing to spend time near low or high densities of kin or non-kin may be critical to ensuring the survival of a cannibalistic species such as the fire salamander at the larval stage.

The fire salamander is an ideal model system for testing associations involving varying aggression levels and variable genetic similarity [2]. In a recent study, we found that fire salamander larvae of lower genetic similarity were more aggressive toward one another [2]. Therefore, we used this system to study whether kinship (i.e. siblings or non-siblings) and the density of conspecific groups affect where and with whom the larvae prefer to allocate their time. Conspecific responses toward different kinship levels and densities may have a crucial impact on species that inhabit small, temporary habitats (e.g. ephemeral pools) and demonstrate high levels of aggression (e.g. cannibalism) [22]. These responses may therefore affect the growth and survival of the species in question [24–26]. Specifically, we tested two novel hypotheses regarding the preferences of individual salamander larvae to stay near different compositions of conspecific groups. The first, “aggression reduction” hypothesis, suggests that an individual larva will spend more time near low densities of conspecific groups and will prefer the presence of its siblings to minimize any potential negative social interactions such as aggression (because non-siblings are more aggressive toward one another than siblings). The second “group advantages” hypothesis suggests that an individual larva will spend more time near high densities of conspecific groups if these conspecifics may be considered prey or there are other advantages of staying in a group (e.g. dilution effects and information centers).

As these fire salamander larvae can assess the degree of their genetic similarity to other larvae in the pool [2], we predicted no or lower aggression between more genetically similar individuals and higher aggression between individuals as the genetic similarity between them declines. To test these two hypotheses, we observed focal larvae and assessed the time they spent near different groups of conspecifics and aggression they directed toward these groups, which varied in density and relatedness (i.e. siblings of the focal larva or its non-siblings).

## Methods

### Ethics statement

Permission to use the salamanders was granted by Israel Nature and Parks Authority (permit 2009/32200). The experiment complied with the current animal protection laws of Israel and was conducted according to the University of Haifa's Animal Experimentation Ethics Committee (permit 120/08).

### Collection and maintenance of animals

During rainy nights in the winter (February 2015), we collected 19 gravid females (see details below) from one breeding site (a pond) in Mount Carmel, northern Israel. Each female was placed in an individual plastic container (30x30x30 cm) filled with shallow water (4 cm depth) and was brought to the laboratory within a few hours following their capture to minimize any potential stress. Females were placed in a climate-controlled room (12L:12D, 22°C) and were fed daily *ad libitum* with *Daphnia* sp. and mosquito larvae. Each female was kept in an individual aquarium (23×35×20 cm) filled with 4 L of aged tap water and stones to allow the female to rest above the water. The water was shallow enough to prevent the stratification of larvae after they had been larviposited. We observed the females every 20 minutes during the daytime. When we noticed a female larvipositing, we immediately removed all the larvae that were already larviposited from the aquarium (and did not use them in the experiment). From that point on, we collected each new larva that was larviposited and immediately placed it into a separate tub (20x20x20 cm) with aged tap water. Therefore, larvae were not together for more than a few minutes to avoid the development of familiarity between siblings. We defined all larvae from one female as family members. Each larva was assigned a letter for individual identification, among its family members (each family was assigned a number, so the first larva from the first family was recorded as 1A, which was written on its tub). The females were then returned to their original natural pond. Larvae included in the study were fed once a day with similar amounts of *Daphnia* sp., (30 *Daphnia* individuals) at the same time and at a rate consistent with their consumption in nature [2]. At the end of the study, the larvae were returned to the pond where their mothers were collected. At the time of the study, there were no records in Israel of *Batrachochytrium salamandrivorans* (Bsal), a fungal pathogen that has recently caused die-offs in natural populations of the European fire salamander (*Salamandra salamandra*; *Linnaeus 1758*) in Europe. Therefore, our permits had no restrictions on returning salamanders to their natural pond.

### Body mass and size

Experiments began when larvae were 5–8 days old. Before this, we recorded the body mass and body length (snout-vent and snout-tail lengths, measured with calipers) of each larva. Larvae that were tested together in a given trial (5–7 individuals, see details below) were matched in size (mean±S.D size of larvae at 5–8 days old were: body mass 0.331 ± 0.008 g; snout-vent length: 1.924 ± 0.044 cm; snout-tail length: 3.677 ± 0.047cm). Therefore, there were no significant differences in body mass or body length among these larvae (*p* values > 0.05 in *t*-tests comparing each focal larva and conspecific larvae tested in the same trial, see details below).

### Experimental set-up

**Fig 1:**
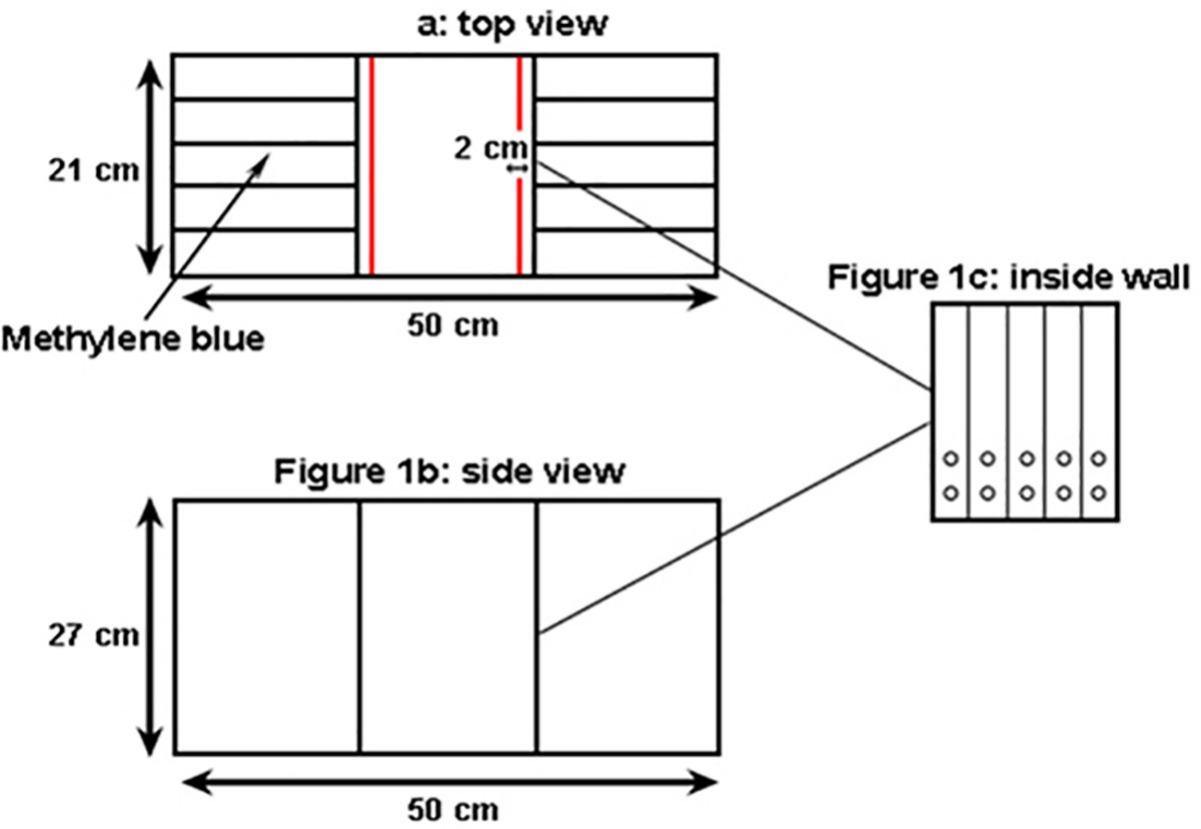
The experimental glass aquarium that was used in the experiment, (1a) top view and (1b) side view. The aquarium consisted of a central chamber and two side chambers. The side chambers were divided into five cells (1a). Each of the two walls between the central and the side chambers had two holes within each of the five cells (each hole 10 mm diameter; 1c), to allow water and chemical flow between the central and the two side chambers. The application of the methylene blue into the central cell is shown.

A transparent glass aquarium (50x21x27 cm; Fig 1A) was filled with 11.55 L of aged tap water. The aquarium was divided into three equal chambers (one central chamber and two side chambers; Fig 1A and 1B). Each of the two side chambers was divided by glass walls into five equal cells (Fig 1A). The two main glass walls separating the central chamber from each of the two side chambers had holes (10 mm diameter) to allow for water passage (Fig 1C).

Chemical cues are known to play a major role during inter- and intra-specific communication in newts and salamanders [27], including larvae, sub-adult and adult fire salamanders [27–29]. Therefore, to ensure that the experimental apparatus also allowed for the transfer of potential chemical cues originating from the larvae (as was found in [28]; in addition to visual cues), we estimated the diffusion rates within the aquarium. We have done so by adding methylene blue (500 μL of 10 mM aqueous solution) to the middle cell of the aquarium’s left side chamber (see details in Fig 1A). We gently mixed the water column at the beginning of the trial to mimic larval movement that may circulate chemical cues. We then collected three water samples at each time point (0, 10, 20, 30, 40 and 50 minutes from the introduction of the methylene blue). We collected the water samples from the left side chamber (2 cm from the methylene blue application point; Fig 1A) and the central chamber (2, 4, 6, and 8 cm away from the glass divider). Namely, we sampled all the away from the glass wall divider and halfway along the central chamber, where we later recorded the behavior of the focal larvae (see experimental procedure for details). The appearance of methylene blue in these water samples was recorded (using Genesys 20 Spectrophotometer, Thermospectronic, Model 4001/4, USA) as absorbance at 640 nm, the maximum absorbance of methylene blue. We repeated this procedure by adding the methylene blue to the right side chamber of the aquarium.

Absorbance at 640 nm was maximal in most middle chamber locations 20 minutes after applying the methylene blue to the middle side cell in either side chamber. This result suggests that the pre-test period of 20 minutes, which allowed larvae time to adjust to the aquarium (see details in the experimental procedure section), was enough for any potential chemical cues to diffuse from the side chambers to the central chamber.

### Experimental procedure

Each experimental trial consisted of one focal larva located in the central chamber, with its non-siblings of the same size in one side chamber and its siblings of the same size in the opposite side chamber, presented at varying densities. We housed only one conspecific larva in any given side cell such that each neighbor was also separated from each other. For the unity of the experiments, each of the non-sibling groups was comprised of larvae from a single mother.

The larvae were observed and their behavior recorded from a distance of about 30 cm from the aquarium. The larvae did not seem to be affected by the presence of the observer, and they kept their apparently normal behavior, as was observed from a greater distance. We observed the behavior of a central focal larva, with conspecific larvae in the two side chambers simultaneously, in the following combinations of density and kinship (described below as siblings or non-siblings of a focal larva):

Experiment A: 3 siblings in one side chamber and 3 non-siblings in the other side chamber

Experiment B: 3 siblings in one side chamber and 1 non-sibling in the other side chamber

Experiment C: 1 sibling in one side chamber and 3 non-siblings in the other side chamber

Female fire salamanders in our study site (i.e. Mount Carmel) were found to larviposit on average 112 ± 43.189 larvae in winter [18]. However, a female fire salamander usually distributes its larvae among a few pools/puddles [30]. Therefore, temporary pools and even more so small puddles may contain one or more larvae. Thus, including five to seven larvae at a time in each trial is a decision that replicates the natural densities of larvae in small water bodies of similar size to our aquarium.

The rationale behind conducting these three experiments was to assess whether given similar densities, focal larvae would prefer to be associated with and aggressive toward non-siblings (experiment A; following findings in Markman et al., 2009). Further, we wanted to test whether, in cases where there are more siblings (experiment B) or more non-siblings (experiment C), focal larvae would be more sensitive to density or kinship—that is, to test the combined effects of density and kinship. Other experimental combinations were possible. However, we were limited in the number of larvae that we could use in experiments due to the limited number of females that we were permitted to collect by Israel Nature and Parks Authority.

We ran nine trials for each of the three experiments, using different combinations of larvae in each trial and testing each larva only once and only in one experiment, to avoid pseudoreplication. Accordingly, we did not use the focal larvae as part of the sibling or non-sibling groups, and vice versa.

Each trial started with a pre-test period of 20 minutes, allowing the larvae to become acclimated to the experimental aquarium (see justification for this time period in the experimental set-up section). We then proceeded to a 30-minute test period, during which we observed the focal larva. Following the test period, we moved each of the larvae to an individual tub and washed the aquarium. Following this cleaning procedure (which took about an hour), we returned the larvae to the experimental aquarium but, this time, to the opposite side chambers (right or left) to account for any potential side bias in the focal larva's behavior. We again allowed the larvae a pre-test period of 20 minutes followed by a 30-minute test period. One experimenter placed the larvae in the aquarium and, therefore, knew their mothers' identities and the relatedness to the focal larva, while a second experimenter acted as a naïve observer, unaware of larvae relatedness, and recorded their behavior.

During the test period, we recorded the time that the focal larva spent within 2 cm of the divider between the central chamber and each of the side chambers, at which point the larva was considered to be near its conspecifics. Further, we recorded the time only when both the focal larva and its neighboring larva stuck on the glass opposite to each other and started synchronically moving in parallel to each other. We also recorded how many bite attempts the focal larva made toward the larvae in the side cells. A bite attempt was distinguished from non-biting movements toward the side cells by an opening and closing of the mouth.

### Statistical analysis

We had nine trials (n = 9) in each experiment (A, B, C). However, we lost data for one trial in experiment C, leaving eight trials (n = 8) for analysis. We used a *sign test* (two-tailed; significant *p* value < 0.05) to test for the effect of the presence of sibling(s) or non-sibling(s) in each of the two side chambers on the time spent by the focal larva near its conspecifics and the number of bite attempts the focal larva directed toward these conspecifics. We ran this analysis separately for each of the three experiments. For example, in experiment B, we compared the time spent by the focal larva near three siblings in one side chamber or near one non-sibling in the other side chamber. The time that the focal larva spent near the varying densities of siblings and non-siblings represented its overall response toward these conspecifics. This response could have resulted from more time spent near conspecifics when they were in groups of three. Therefore, we were interested to learn if there was a difference in the focal larva's time allocation per neighboring larva in each of the three experiments. To indirectly standardize the effects of the two groups of conspecifics, we divided the time spent by the focal larva near each of the two side chambers by the number of larvae in each of the side chambers (i.e. by three for groups of three larvae). The overall time spent by the focal larva near the group of conspecifics and the time spent per neighboring larva are not independent of each other but are pertinent to different points of interest. For examples of this idea see [31–32]. For instance, by calculating the mean time spent by the focal larvae near each neighboring conspecific, we could tease apart the effects of density and kinship to some extent (see the discussion for more details). We also divided the total bite attempts made by the focal larva by the number of larvae in each of the side chambers (i.e. by three for groups of three larvae). We could have standardized the data by dividing the mean number of bite attempts directed per larva by the mean time spent near a neighboring larva to calculate the rate of bite attempts made by the focal larva. However, we were interested in the gross response of the focal larva and not in the rate at which it tried to bite the neighboring larva.

## Results

### Total time spent near siblings or non-siblings

**Fig 2:**
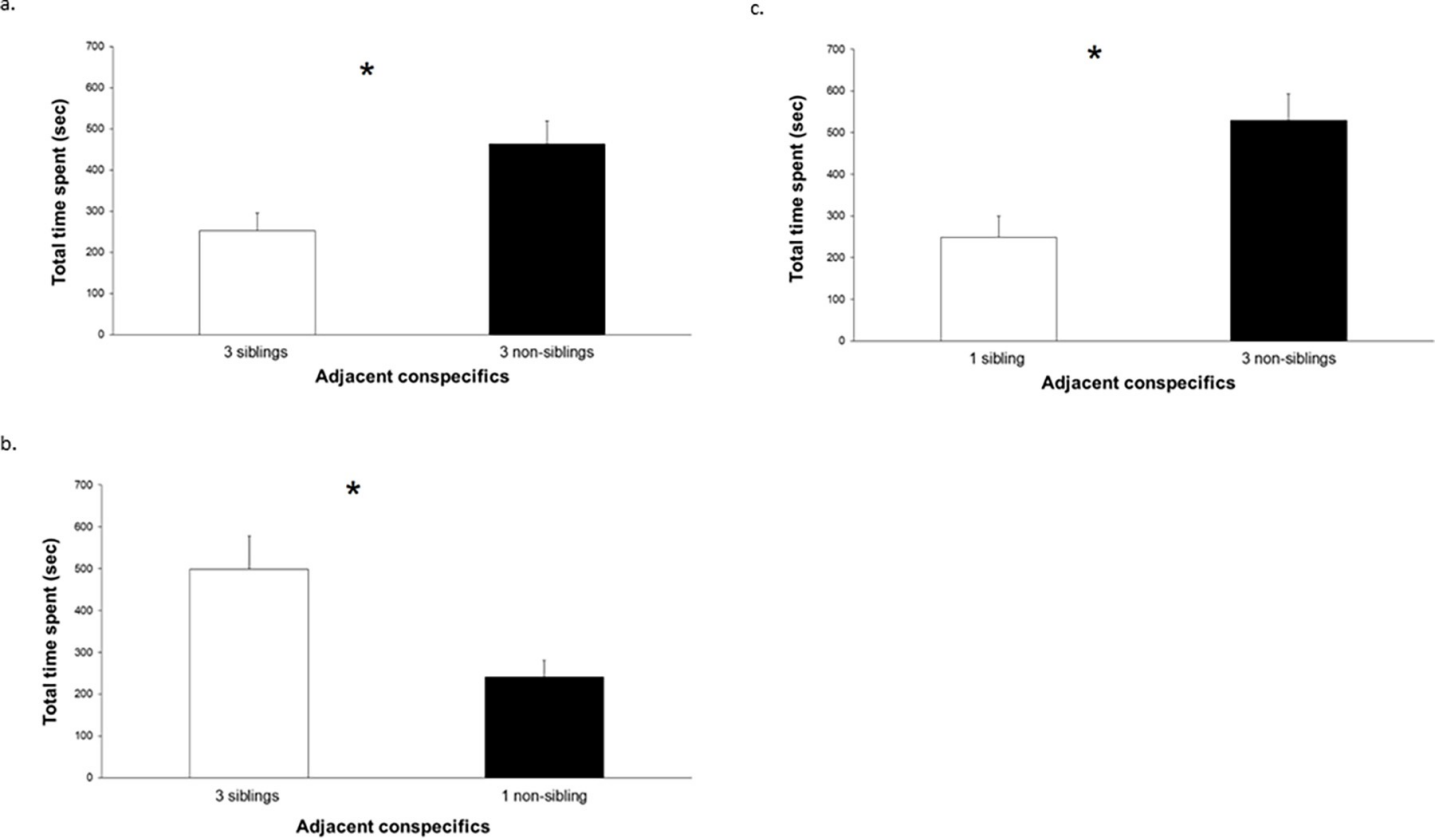
Total time spent (in seconds; mean ± s.e.) by a focal larva during 30 min sessions, in the experimental aquarium, within 2 cm away from adjacent conspecifics, when they were in: (a) Experiment A: three siblings in one side chamber versus three non-siblings in the other side chamber, (b) Experiment B: three siblings in one side chamber versus one non-sibling in the other side chamber, and (c) Experiment C: one sibling in one side chamber versus three non-siblings in the other side chamber. The side chamber in which these conspecifics were housed within each of the three experiments was randomly alternated between the right and left chambers. In each of the three experiments, mean is shown for each of the two groups of conspecifics averaged across the two side chambers (right or left). An asterisk (*) denotes a significant difference (P < 0.05; *sign test*) between the time spent near the two groups of conspecifics.

In experiment A, kinship affected the behavior of the focal larva as the total time that the focal larva spent near the group of three non-siblings was significantly longer than the time it spent near the group of three siblings (*sign test*: *p* = 0.031; Fig 2A). In experiment B, the total time that the focal larva spent near the group of three siblings was significantly longer than the time it spent near the one non-sibling *(sign test*: *p* = 0.031; Fig 2B). In experiment C, the total time that the focal larva spent near the group of three non-siblings was significantly longer than the time it spent near the one sibling (*sign test*: *p* = 0.004; Fig 2C). So, the focal larvae spent more time near the higher density of conspecifics irrespective of kinship level (experiments B and C; i.e. when there were unequal numbers of siblings and non-siblings).

### Time spent near a sibling or a non-sibling

In experiment A, kinship affected the behavior of the focal larva as the mean time that the focal larva spent near a neighbor, when there were three non-siblings in the side chamber, was significantly longer than the time it spent near each neighboring larva, when there were three siblings (*sign test*: *p* = 0.031). In experiment B, the mean time that the focal larva spent near a neighboring larva, when there were three siblings in the side chamber, was not significantly different from the time it spent near one non-sibling (*sign test*: *p* = 0.238). Similar results were obtained in experiment C, when there were three non-siblings in one side chamber and one sibling in the other side chamber (*sign test*: *p* = 0.454). Therefore, the time spent per neighboring larva was not affected by kinship in experiments B and C. That is, when the densities of siblings and non-siblings were different, the focal larva spent similar amounts of time per neighboring larva whether it was a sibling or a non-sibling.

### Total number of bite attempts directed toward siblings or non-siblings

**Fig 3:**
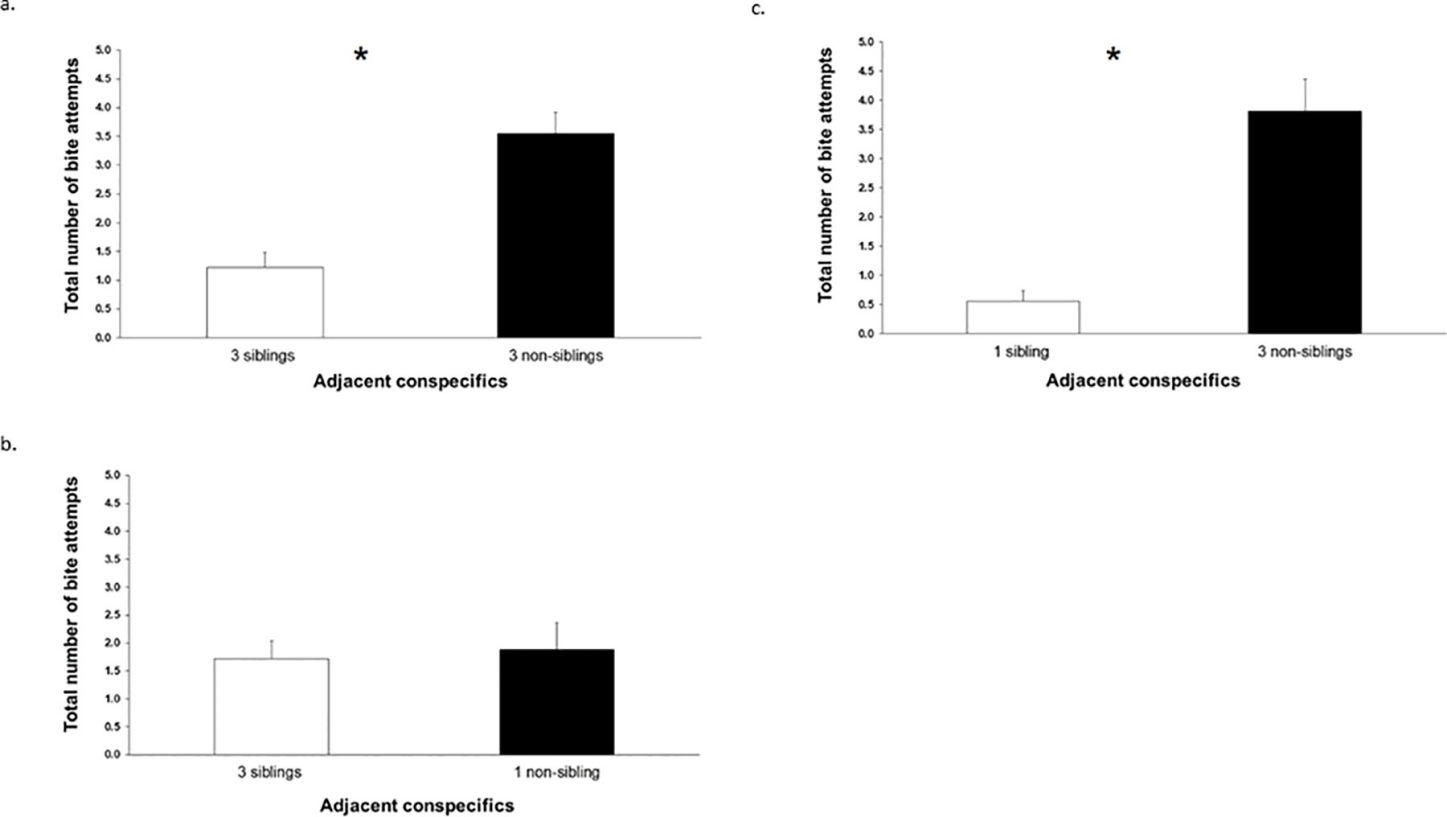
The total number of bite attempts (mean ± s.e.) directed by a focal larva toward adjacent conspecifics during 30 min sessions, in the experimental aquarium, within 2 cm away from these conspecifics, when there were in: (a) Experiment A: three siblings in one side chamber versus three non-siblings in the other side chamber, (b) Experiment B: three siblings in one side chamber versus one non-sibling in the other side chamber, (c) Experiment C: one sibling in one side chamber versus three non-siblings in the other side chamber. In each of the three experiments, total is shown for each of the two groups of conspecifics averaged across the two side chambers (right or left). An asterisk (*) denotes a significant difference (P < 0.05; *sign test*) between the number of bite attempts directed toward the two groups of conspecifics.

In experiment A, there was a significant effect of kinship on the number of bite attempts demonstrated by the focal larva. That is because the total number of bite attempts that the focal larva directed toward the group of three non-siblings was significantly higher than the number of bite attempts it demonstrated toward the group of three siblings (*sign test*: *p* = 0.001; Fig 3A). In experiment B, there was no significant difference in the total number of bite attempts that the focal larva directed toward the group of three siblings or one non-sibling (*sign test*: *p* = 0.549; Fig 3B). In experiment C, the total number of bites that the focal larva directed toward the group of three non-siblings was significantly higher than toward the one sibling (*sign test*: *p* < 0.001; Fig 3C).

### Number of bite attempts directed toward a sibling or a non-sibling

In experiment A, the mean number of bite attempts that the focal larva directed toward a neighboring larva among a group of three non-siblings was significantly higher than the number of bite attempts it directed toward a neighboring larva among a group of three siblings (*sign test*: *p* = 0.001). In experiment B, the mean number of bite attempts that the focal larva directed toward a neighboring larva among a group of three siblings was significantly lower than the number of bite attempts it directed toward a single non-sibling larva (*sign test*: *p* = 0.013). In experiment C, the mean number of bite attempts that the focal larva directed toward a neighboring larva among a group of three non-siblings was significantly higher than the number of bite attempts it directed toward a single sibling (*sign test*: *p* = 0.022). To summarize, in all three experiments, the focal larvae directed significantly more bite attempts, per neighboring larva, toward a non-sibling than a sibling.

## Discussion

In this study, we tested the response of focal larvae to both the density and relatedness of conspecifics. We changed the densities and relatedness of the conspecifics in relation to the focal larvae. We tested two hypotheses: “aggression reduction” and “group advantages”. Our results suggest that density was the main factor that affected the focal larvae’s time allocation in experiments B and C (in which the densities of siblings and non-siblings were unequal). Only at equal densities of siblings and non-siblings (experiment A) did kinship affect the behavior of the focal individuals, as the focal larvae spent more time near and were more aggressive toward their non-siblings as compared to their siblings. Thus, these results support the "group advantage" hypothesis: the focal larvae preferred higher densities of conspecifics and both spent more time near and were more aggressive toward non-siblings when densities of sibling and non-sibling groups were equal (all of which was predicted by the "group advantage" hypothesis). These results do not support the "aggression reduction" hypothesis, where we would expect the focal larvae to spend more time near their siblings and near lower density of conspecifics.

Following the logic of the "group advantage" hypothesis, our findings may be explained by the advantages of staying with a larger group and/or because these cannibalistic larvae may view a high density group of conspecifics as potential food, especially when non-siblings are present [22]. While a focal larva may be attacked by its conspecifics, this risk seemed to be accepted by focal larvae in our study—and in nature too, when larvae approach each other and may attack or be attacked by conspecifics. Attacks may occur even between larvae of the same body size (as observed by Sadeh and Markman, personal observations) [2]. Whether or not the focal larvae had an intention to feed on other larvae, by definition, staying near more conspecifics potentially increase the number of individuals that may be attacked or eaten. Additionally, spending more time with unrelated conspecifics by itself may result in more intensive agonistic behaviors such as bite attempts. There is an evidence that aggression and cannibalism in fire salamanders is size and resource dependent [20, 21, 33–35]. This may suggest that the observed aggression attempts could also represent spacing mechanisms between conspecifics.

Thus, the benefit of staying with a large group because it provides potential food is only an advantage for individuals that are large enough to consume other conspecifics or parts of their conspecifics. Joining a group may be a potential disadvantage for small individuals that might be eaten by larger conspecifics in groups of varying body sizes. However, in the present study, we wanted to control for body size differences and assess whether individuals of the same size share similar behavioral responses. Additionally, the preference to stay with groups versus with an isolated individual may be explained by the stronger olfactory and visual stimulation offered by groups than by a single individual. Another possible explanation is the fact that many animals, especially those that are strongly water-dependent, aggregate to reduce desiccation or even to regulate their temperature [36].

Our results demonstrate that focal larvae were more aggressive toward non-sibling larvae, irrespective of whether the non-sibling was in a group or alone. Similarly, in a previous study without varying densities, we found that focal larvae were more aggressive toward non-siblings than siblings [2]. In that study, we found differential aggressiveness among larvae along a broad within-species genetic relatedness continuum, at various levels of genetic similarity within and across pools and populations where the mothers had been collected (that is, siblings or non-siblings from the same pool, close populations, and distant populations) [2]. Markman et al. [2] found that bites occurred most frequently between larvae from distant populations, with the lowest average number of bites among siblings [2]. Such aggression may result in cannibalism, which may help to explain the fact that the focal larvae in the current study spent more time near non-siblings when they were present at the same density as siblings. Furthermore, focal larvae were always more aggressive toward non-sibling larvae than sibling larvae.

Sadeh [22] compared siblings and genetically mixed larval cohorts in fire salamanders throughout their larval period. He found that the rates of cannibalism were higher in mixed cohorts, resulting from higher aggression toward non-siblings. Nevertheless, overall survival before habitat loss (e.g. when the pond dried out) was similar between treatments (sibling or mixed cohorts). The probability that a surviving larva would metamorphose before habitat loss early in the season was higher in the mixed cohorts than sibling cohorts, partly due to these higher rates of cannibalism in the mixed cohorts [22]. A larva that cannibalizes another larva will typically develop at a faster rate and metamorphose before non-cannibalistic larvae. In the present study, we used only individuals of the same body size and cannibalism was restricted by the glass divider that separated focal larvae from their conspecifics. Despite similar body sizes, the focal larvae were still aggressive toward their conspecifics (even though we fed our larvae on a regular basis). Accordingly, larvae may greatly benefit from cannibalistic acts irrespective of their hunger levels. Further, Michimae and Wakahara [37] observed that the cannibalistic acts of the salamander *Hynobius retardatus* occurred in both low and high food supply. However, the frequency of cannibalism was significantly higher at low than at high levels of food supply, and in mixed sibling groups than in the pure sibships.

The experimental aquarium had a small volume of water, resembling a small, temporary pond, where adult salamanders usually larviposit in nature [2]. Under such conditions, the aggression of the larvae toward each other increases [22]. Indeed, the current study demonstrated that the time allocation and aggressive behavior of the focal individuals were usually directed toward higher density of neighbors, especially if they were non-siblings, as they may serve as a potential food source. Therefore, an individual in a cannibalistic system will likely benefit from being associated with a group of non-siblings (of the same or smaller size) to take advantage of full cannibalism (i.e. consuming a whole larva) or partial cannibalism (i.e. taking part in group cannibalism or consuming a body part of another larva; as was observed by Sadeh and Markman personal observations). Sadeh et al. [38] stated that especially exploitative competition for invertebrate prey items, but also aggression and cannibalism, may occur between larvae of the same cohort, namely of the same age and similar size, in a pond. This especially holds true when the pond is about to desiccate and/or there is a high level of competition among the larvae—that is, when there are time and food constraints on metamorphosis and survival rates. Indeed, Sadeh [22] found that high genetic relatedness and a lack of cannibalism may have severe consequences for all of the larvae in a puddle under severe competitive conditions.

However, we cannot rule out further advantages for the focal larvae to stay with a group. Additional advantages of staying with a group may include dilution effects; confusion effects, when escaping from predators; group defense; higher vigilance by more group members; and the group as a center of information [14–15, 39–41]. All of these benefits of group living should potentially increase individual fitness [42]. The possible costs associated with being in a group may include competition around resources, contraction of pathogens, and attracting predators [43]. However, if the benefits are greater than the costs, joining a group will be more attractive [13].

The dilution effects might be important in the presence of predators like the white-throated kingfisher (*Halcyon smyrnensis; Aves*) (Sadeh, personal observations) and the dice snake (*Natrix tessellate*); both species are common in Israel and feed on salamander larvae [44]. Further, the larvae in our study were of similar body sizes, which may, in part, explain their aggregation toward conspecifics to ensure potentially higher levels of vigilance against these predators. Other possible explanations for aggregation among similar sized larvae include a lower chance of suffering from cannibalism by larger conspecifics and higher foraging efficiency in some instances (e.g. when prey items are abundant enough and can perform predator-confusion effects).

To summarize, group size and kinship can influence whether a larva will spend more time with conspecifics, depending on the net benefits of joining a larger group and/or staying near non-siblings. Joining a larger group may increase potential interactions, such as aggression, between individuals. The aggression of a focal larva may be explained by various factors like group size, relatedness, hunger levels, pond size and its likelihood to dry out, predation risk and food availability including the chances of performing cannibalistic acts.

Taken together, we found that larvae spent more time near higher density of conspecifics, irrespective of kinship, and more time near non-siblings, if groups of non-siblings and siblings were present at the same density. Moreover, focal larvae demonstrated more aggression toward non-sibling larvae. These findings support the “group advantages” hypothesis. These results do not prove the "aggression reduction" hypothesis, where we would expect a focal larva to spend more time near its siblings and near lower density of conspecifics. It should be noted that we were limited in the number of larvae that we were allowed to collect by the Nature Reserve Authority. Therefore, in order to generalize our findings even more, we hope to address each of these explanations by using larger sample sizes of larvae in future studies.

We suggest that future studies should test the combined effects of food availability, conspecific group density, and the larvae’s developmental stage, body state (i.e. starvation levels and body energy balance), and body size on the tendency to spend time near and be aggressive toward individuals of varying levels of genetic relatedness. Such comprehensive studies would encapsulate as many as possible ecological and physiological determinants of group living.
